# Supplemental Nutrition Assistance Program and Adherence to Antihypertensive Medications

**DOI:** 10.1001/jamanetworkopen.2023.56619

**Published:** 2024-02-23

**Authors:** Md. Mohaimenul Islam, Ximena Oyarzun-Gonzalez, Seuli Bose-Brill, Macarius M. Donneyong

**Affiliations:** 1 Outcomes and Translational Sciences, College of Pharmacy, The Ohio State University, Columbus; 2 Division of General Internal Medicine, Department of Internal Medicine, The Ohio State University College of Medicine, Columbus

## Abstract

**Importance:**

Nonadherence to antihypertensive medications is associated with uncontrolled blood pressure, higher mortality rates, and increased health care costs, and food insecurity is one of the modifiable medication nonadherence risk factors. The Supplemental Nutrition Assistance Program (SNAP), a social intervention program for addressing food insecurity, may help improve adherence to antihypertensive medications.

**Objective:**

To evaluate whether receipt of SNAP benefits can modify the consequences of food insecurity on nonadherence to antihypertensive medications.

**Design, Setting, and Participants:**

A retrospective cohort study design was used to assemble a cohort of antihypertensive medication users from the linked Medical Expenditure Panel Survey (MEPS)–National Health Interview Survey (NHIS) dataset for 2016 to 2017. The MEPS is a national longitudinal survey on verified self-reported prescribed medication use and health care access measures, and the NHIS is an annual cross-sectional survey of US households that collects comprehensive health information, health behavior, and sociodemographic data, including receipt of SNAP benefits. Receipt of SNAP benefits in the past 12 months and food insecurity status in the past 30 days were assessed through standard questionnaires during the study period. Data analysis was performed from March to December 2021.

**Exposure:**

Status of SNAP benefit receipt.

**Main Outcomes and Measures:**

The main outcome, nonadherence to antihypertensive medication refill adherence (MRA), was defined using the MEPS data as the total days’ supply divided by 365 days for each antihypertensive medication class. Patients were considered nonadherent if their overall MRA was less than 80%. Food insecurity status in the 30 days prior to the survey was modeled as the effect modifier. Inverse probability of treatment (IPT) weighting was used to control for measured confounding effects of baseline covariates. A probit model was used, weighted by the product of the computed IPT weights and MEPS weights, to estimate the population average treatment effects (PATEs) of SNAP benefit receipt on nonadherence. A stratified analysis approach was used to assess for potential effect modification by food insecurity status.

**Results:**

This analysis involved 6692 antihypertensive medication users, of whom 1203 (12.8%) reported receiving SNAP benefits and 1338 (14.8%) were considered as food insecure. The mean (SD) age was 63.0 (13.3) years; 3632 (51.3%) of the participants were women and 3060 (45.7%) were men. Although SNAP was not associated with nonadherence to antihypertensive medications in the overall population, it was associated with a 13.6–percentage point reduction in nonadherence (PATE, −13.6 [95% CI, −25.0 to −2.3]) among the food-insecure subgroup but not among their food-secure counterparts.

**Conclusions and Relevance:**

This analysis of a national observational dataset suggests that patients with hypertension who receive SNAP benefits may be less likely to become nonadherent to antihypertensive medication, especially if they are experiencing food insecurity. Further examination of the role of SNAP as a potential intervention for preventing nonadherence to antihypertensive medications through prospectively designed interventional studies or natural experiment study designs is needed.

## Key Points

**Question:**

Is receipt of Supplemental Nutrition Assistance Program (SNAP) benefits associated with nonadherence to antihypertensive medications and, if so, does this association vary by food insecurity status?

**Findings:**

In this cohort study with 6692 participants, receipt of SNAP benefits was associated with a nearly 14–percentage point reduction in nonadherence to antihypertensive medications among food-insecure patients with hypertension but not among their food-secure counterparts.

**Meaning:**

These findings suggest that SNAP should be further investigated as a potential intervention for preventing nonadherence to antihypertensive medications, especially among food-insecure individuals.

## Teaser

This cohort study examines the association between receipt of Supplemental Nutrition Assistance Program (SNAP) benefits and adherence to antihypertensive medications.

## Introduction

Nearly half of US adults (approximately 121.5 million [47.3%]) have hypertension, which contributes to about 1000 deaths daily.^1,2^ Hypertension is a strong modifiable risk factor for cardiovascular diseases (CVDs), including coronary heart disease and stroke, which are the 2 leading causes of death in the US.^3,4^ The economic burden of hypertension is substantial for patients, health care systems, and countries, due to the loss of productivity from morbidity and premature death.^5,6^ According to the American Heart Association, annual health care costs (both direct and indirect) for the US population with hypertension are between $131 and $198 billion.^7^ Pharmacologic treatment with antihypertensive medications is the main approach to managing hypertension when nonpharmacologic interventions, such as physical activity, consumption of a healthy diet, and alcohol reduction, are unable to control hypertension in a timely fashion.^8,9^

Nonadherence to antihypertensive medications (ie, the extent to which patients do not use antihypertensive medications per their clinician’s recommendation) is also highly prevalent.^10,11,12^ The World Health Organization (WHO) multidimensional framework of medication adherence identifies numerous risk factors^13^ at the following levels: patient (eg, depression, forgetfulness, and perceptions of antihypertensive medication efficacy), socioeconomic status (eg, health-related social needs such as food insecurity, lack of social support, and lack of transportation), condition (eg, chronic treatment duration and symptom severity), therapy (eg, long duration of treatment, severe adverse events, and high copays for medications), and health care system (eg, poor access to health care, poor drug supply, and poor patient-clinician communication).^14,15,16^

Although barriers to antihypertensive medication adherence remain multifactorial and complex, modifiable health-related social needs, such as food insecurity, are emerging as potential modifiable targets for improving adherence. Food insecurity impairs medication adherence because patients are confronted with a challenging situation in which they must either feed themselves or spend limited resources on antihypertensive medications.^17,18,19,20^ Often, it is patients of limited financial means who must make this difficult choice.

The Supplemental Nutrition Assistance Program (SNAP) is the largest social intervention program in the US that provides financial assistance to low-income families through vouchers that can only be applied toward purchasing food.^21^ Some analyses have reported that SNAP participation may potentially reduce poverty by as much as 16%—equivalent to 8 million people—in the US.^22,23^ In addition, SNAP is an effective intervention for reducing food insecurity, as reported in a national study in which SNAP benefits were associated with an up to 30% reduction in food insecurity.^24^ Given that SNAP intervention is effective in reducing both poverty and food insecurity, which are both major risk factors for medication nonadherence, it is plausible that SNAP may be effective in preventing nonadherence to antihypertensive medications. In a recent analysis of patients with diabetes, researchers found that SNAP benefit receipt was associated with lower rates of cost-related medication nonadherence.^17^ In this analysis, we evaluated the association between SNAP benefit receipt and nonadherence to antihypertensive medications, and we further investigated the role of food insecurity in this association.

## Methods

This cohort study was approved by The Ohio State University Institutional Review Board. Informed consent was waived because publicly available data were used. The study followed the Strengthening the Reporting of Observational Studies in Epidemiology (STROBE) reporting guideline.

### Conceptual Framework

**Figure 1.**
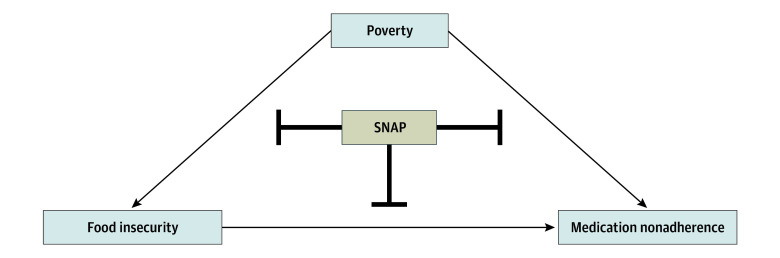
Conceptual Framework for Medication Nonadherence SNAP indicates Supplemental Nutrition Assistance Program.

The associations among poverty, food insecurity, nonadherence, and SNAP are highlighted through a conceptual framework in Figure 1. In this framework, we hypothesized that poverty may cause nonadherence to antihypertensive medications either directly or indirectly via food insecurity. Given that SNAP is an effective intervention for reducing both poverty and food insecurity, SNAP may potentially block the direct and indirect pathways through which poverty may lead to nonadherence as shown in Figure 1.

### Data Source

For this analysis, we linked the household component of data from the Medical Expenditure Panel Survey (MEPS) for 2016 to 2017^25^ and the National Health Interview Survey (NHIS) dataset.^26^ Linkage between the MEPS and the NHIS was performed by the MEPS investigators prior to sharing the linked MEPS-NHIS files. These files also included a crosswalk, which we used to merge the MEPS full-year public use files and the NHIS person-level public use data files.^27^ At the time of completing the current analysis in 2020, the MEPS data files were updated up to 2018; within these data files, only the 2016 to 2017 files included food insecurity data. The MEPS–health care data are collected from a nationally representative sample of households through an overlapping panel design (eAppendix 1 in Supplement 1); these data are collected in 5 rounds of interviews during a 2-year period. Survey questionnaires are used to gather data regarding several factors on health care utilization and expenditure, including but not limited to self-reported health status, medical conditions, health insurance status, health care access, and prescription medication use. Similar to the MEPS, the NHIS is a nationally representative cross-sectional survey of US households that aims to collect health information including, but not limited, to SNAP participation status, demographic, socioeconomic factors, health status, health care access, and health behavior.

### Study Design

We applied a retrospective cohort study design to assemble a cohort of antihypertensive medication users from the linked MEPS-NHIS dataset (eAppendix 1 in Supplement 1). We linked the MEPS and NHIS datasets for the analyses because although information on medication refill adherence (MRA), food insecurity, and several other important covariates of interest was captured in the 2016 to 2017 MEPS datasets, information regarding SNAP receipt status was assessed in only the NHIS data. All baseline covariates and effect modifiers (food insecurity status) were measured using data captured during the round 1 surveys from each data cycle, 2016 and 2017, respectively. For each survey year, participants were asked to report whether they had received SNAP any time during the prior year (eg, responses on SNAP receipt status in 2016 were in reference to whether a participant had received SNAP in 2015). The eFigure in Supplement 1 presents the initial sample of participants, those excluded because of restrictions, and the final sample used for analysis.

### Outcome

The following detailed information on prescription medications was obtained directly from pharmacies after MEPS investigators had obtained consent from respondents: payments, payers, date each prescription was filled, quantity dispensed, and the National Drug Code of dispensed medications. The MEPS investigators also obtained from pharmacies information regarding the number of times that medications were filled by a MEPS participant within a given calendar year.^28^ We identified antihypertensive medication users through the therapeutic class codes corresponding to any of the antihypertensive agents listed in the eTable in Supplement 1. Based on information regarding the frequency and quantity of antihypertensive medication dispensed, we calculated MRA for antihypertensive medication use based on the following formula, which is widely used for measuring refill adherence^29,30,31^:
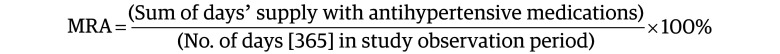
The mean number of MRAs per antihypertensive medication therapeutic class was calculated for patients who used multiple antihypertensive medications. If a patient’s MRA was less than 80%, they were classified as nonadherent to antihypertensive medications.

### Assessment of SNAP Status

In the NHIS, SNAP receipt status was assessed through a single question: “Did you or anyone in your family get benefits from SNAP in the past 12 months?” Individuals who responded positively to this question were classified as SNAP recipients, whereas those who did not were classified as nonrecipients.

### Assessment of Food Security Status

Food insecurity status was assessed in the 2016 to 2017 MEPS cycles by using the 10-item US Department of Agriculture (USDA) Food Security Survey in reference to the past 30 days prior to the survey.^32,33,34^ For example, respondents were asked “how often in the last 30 days anyone in the household worried whether food would run out before getting money to buy more” or “how often in the last 30 the food purchased didn’t last and the person/household didn’t have money to get more.” Responses to these questions were summed to create a score ranging from 0 to 10 for each MEPS participant. Individuals with scores of 1 or greater were considered food insecure, and those who responded negatively to all those items were considered food secure, which is the standard definition of food insecurity and food security based on the USDA Food Security Survey.^34,35^

### Potential Confounders

**Figure 2.**
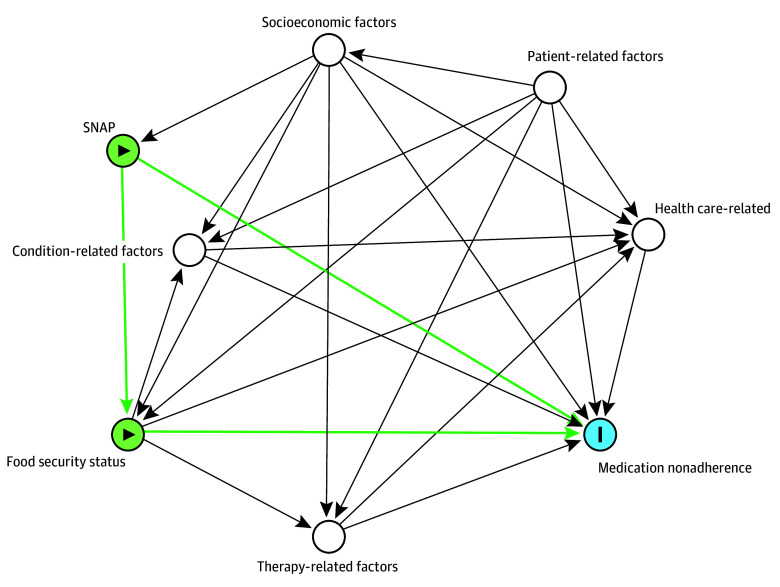
Directed Acyclic Graph for Identifying Confounders and Effect Modifiers of the Associations Between Supplemental Nutrition Assistance Program (SNAP) Benefit Receipt and Medication Nonadherence Green circles are exposures; the blue circle is the outcome variable.

We applied the WHO multidimensional framework of medication adherence to guide the selection and inclusion of several covariates as potential confounders.^13^ Several individual patient, health care practitioner, and health care system factors are identified in the WHO framework as determinants of medication adherence. We applied a directed acyclic graph (Figure 2) to identify and visualize the hypothesized relationships between SNAP, food insecurity, and nonadherence to antihypertensive medications. Factors considered to be associated with both food insecurity and nonadherence to antihypertensive medications were selected as potential confounders and included several demographic factors, such as age, sex, self-reported race and ethnicity (Hispanic, non-Hispanic Black [hereinafter, Black], non-Hispanic White [hereinafter, White], and other race or ethnicity [including American Indian or Alaska Native, Asian, Native Hawaiian or Other Pacific Islander, unknown race or ethnicity, and other or multiple races or ethnicities]), socioeconomic status (eg, educational attainment and poverty level), health status (eg, poor physical health and functional limitation), and health care access (eg, health insurance and out-of-pocket costs) (Table 1). Race and ethnicity was considered a confounder because Black individuals are significantly more likely to experience food insecurity, receive SNAP benefits, and be nonadherent to antihypertensive medications; additionally, race and ethnicity has been shown to be correlated with poverty, which is a determinant of both SNAP and nonadherence to antihypertensive medications. We created dummy variables for each of the baseline categorical variables for the analyses.

**Table 1.** Distribution of Baseline Covariates by SNAP Status Before and After Inverse Probability Weighting

Baseline characteristic	SNAP status^a^			Absolute standardized difference	
	Total (n = 6692)	SNAP (n = 1203)	No SNAP (n = 5489)	Before	After
**Patient related**					
Age, y					
Mean (SD)	63.0 (13.3)	59.5 (13.9)	63.8 (13.0)	0.3	0
Category					
18-34	156 (2.4)	55 (5.1)	101 (2.0)	0.1	0
35-49	914 (13.3)	218 (19.1)	696 (12.6)	0.2	0
50-64	2374 (34.3)	481 (40.0)	1893 (33.6)	0.2	0
65-79	2452 (38.0)	341 (26.4)	2111 (39.4)	0.5	0
≥80	796 (12.0)	108 (9.1)	688 (12.4)	0.1	0
Sex					
Female	3632 (51.3)	777 (59.7)	2855 (50.1)	0.3	0.1
Male	3060 (48.7)	426 (40.3)	2634 (49.9)	0.2	0.1
Race and ethnicity					
Hispanic	1175 (9.8)	295 (15.7)	880 (8.9)	0.6	0.1
Non-Hispanic Black	1602 (15.2)	445 (28.0)	1157 (13.4)	0.1	0
Non-Hispanic White	3711 (71.6)	416 (50.8)	3295 (74.6)	0.5	0
Other^b^	204 (3.4)	47 (5.5)	157 (3.1)	0.1	0
Born in US					
Yes	5382 (87.1)	893 (80.9)	4489 (88.0)	0.1	0.1
Region					
South	2919 (42.8)	573 (44.2)	2346 (42.6)	0.4	0.1
**Socioeconomic status related**					
Marital status					
Married	3525 (57.9)	334 (30.1)	3191 (61.9)	0.1	0.1
Education					
Up to high school	3425 (52.3)	644 (59.0)	2781 (51.4)	0	0.1
Employment status					
Unemployed	4240 (59.7)	981 (78.2)	3259 (57.0)	0.1	0.1
Food security status					
Food secure	5354 (85.2)	663 (57.1)	4691 (89.2)	0.7	0.1
Food insecure^c^	1338 (14.8)	540 (42.9)	798 (10.8)	0.7	0.1
**Condition related**					
Poor physical health	896 (11.8)	315(27.6)	518 (9.8)	0.4	0
Functional limitation	1701 (21.8)	592 (50.2)	1109 (17.8)	0.6	0
**Health care system related**					
Uninsured	318 (3.4)	73 (6.0)	245 (3.0)	0	0.2
Out-of-pocket cost (>$500/y)	1044 (17.5)	80 (7.5)	964 (18.9)	0.3	0.1

Abbreviation: SNAP, Supplemental Nutrition Assistance Program.

^a^ Values are presented as No. (%) of participants, with unweighted No. values and weighted percentages given.

^b^ Encompasses individuals who identified as American Indian or Alaska Native, Asian, or Native Hawaiian or Other Pacific Islander; declined to disclose race and ethnicity; or identified as other or multiple races or ethnicities.

^c^ Not included in the propensity score model.

### Statistical Analysis

Our analysis focused on addressing potential confounders and estimating the treatment effects of SNAP on medication nonadherence. First, inverse probability weighting techniques were used to address the potential confounding effects of the covariates identified through the directed acyclic graph approach. Propensity scores for each study participant were calculated as the probability of a patient receiving (vs not receiving) SNAP benefits, conditional on all measured baseline covariates; food insecurity was excluded from this model given that we prespecified this variable as an effect modifier. The resultant probabilities were inverted to create inverse probability weights (IPWs), which were then used for weighting the sample to control for measured confounding (eAppendix 2 in Supplement 1). After weighting the sample with IPWs, we applied a standardized difference test to assess the balance of the distribution of baseline covariates between SNAP recipients vs nonrecipients among the inverse probability weighted sample and the original unweighted sample. Second, we estimated the population average treatment effects (PATEs) of SNAP on nonadherence to antihypertensive medications through a probit regression model that was weighted by the product of the IPW, and the MEPS survey weights, in the overall sample. The PATE in this overall sample represents the percentage point difference in nonadherence between SNAP recipients vs nonrecipients irrespective of participant food insecurity status.

We tested whether the association between SNAP receipt status and nonadherence differed between the food-secure and food-insecure subgroups through a prespecified stratified analysis approach.^36^ To do this, we created subgroup-specific propensity scores separately for the food-secure and food-insecure subgroups and we used these scores for weighting to control for the confounding effects of all baseline variables included in the overall propensity score model. Similar to the overall analysis, we next estimated the PATE for each food insecurity subgroup to determine whether food insecurity status modified the associations between SNAP and medication nonadherence. The potential effect modification of food insecurity was tested by evaluating for statistical differences in the magnitude and direction of the PATEs for the food-secure and food-insecure subgroups; PATEs were considered statistically significantly different if their respective 95% CIs did not overlap.

In sensitivity analysis, we evaluated for a potential dose-response association by quantifying the associations between levels of frequency of SNAP benefit receipt and nonadherence to antihypertensive medications. Because SNAP benefits are disbursed on a quarterly basis based on eligibility, an individual who is continuously eligible throughout the year would receive SNAP benefits 4 times (ie, for all 4 quarters of the year). We categorized the frequency of SNAP benefit receipt (1-3, 4-6, 7-9, and 10-12 times per year) and estimated PATEs, overall and by food insecurity status, for each of these categories. The category-specific PATEs were calculated by comparing the rate of nonadherence among each category of SNAP benefit receipt with nonreceipt (0 times per year).

We used Stata, version 14.0 (StataCorp LLC), to implement the statistical analyses as described in this section. Data analysis was performed from March to December 2021.

## Results

This study included 6692 antihypertensive medication users (survey-weighted N = 68 523 236). Their mean (SD) age was 63.0 (13.3) years; 3632 (51.3%) were women and 3060 (45.7%) were men. A total of 1602 participants (15.2%) were Black, 1175 (9.8%) were Hispanic, 3711 (71.6%) were White, and 204 (3.4%) were of other race or ethnicity. There were 1203 participants (12.8%; survey-weighted n = 8 553 051) who had received SNAP benefits. A summary of the distribution of baseline covariates, overall and by SNAP status, is reported in Table 1. Overall, 1338 individuals (14.8%) reported experiencing food insecurity in the past 30 days prior to being surveyed; however, nearly half of the SNAP recipients (540 [42.9%]) reported being food insecure compared with nonrecipients (798 [10.8%]). After the sample was weighted by IPWs, all baseline covariates were balanced between SNAP recipients and nonrecipients.

### Association Between SNAP Receipt and Nonadherence to Antihypertensive Medications

Overall, 3849 antihypertensive medication users (55.8% weighted) were nonadherent during our study period. Table 2 summarizes PATEs of SNAP receipt (vs no SNAP receipt) on nonadherence to antihypertensive medications, generated from the weighted probit regression models as described in the Methods. No difference in nonadherence to antihypertensive medications was observed between SNAP recipients vs nonrecipients (687 [56.4% weighted] vs 3162 [55.8% weighted]; PATE, 1.7 [95% CI, −21.2 to 24.6]). Similarly, the rate of nonadherence to antihypertensive medication was similar (385 [56.7% weighted] vs 2671 [55.2% weighted]; PATE, 5.0 [95% CI, −15.8 to 25.7]) between SNAP recipients and nonrecipients among the 5354 patients who did not report experiencing food insecurity. In contrast, among the subgroup of 1338 antihypertensive medication users who experienced food insecurity, the rate of nonadherence to antihypertensive medications was 8.17 percentage points lower among SNAP recipients compared with nonrecipients (302 [56.0% weighted] vs 491 [60.0% weighted]; PATE, −13.6 [95% CI, −25.0 to −2.3]). The observed PATE estimates among the food-secure vs food-insecure subgroups were statistically significantly different, suggesting that food insecurity modified the association between SNAP and nonadherence to antihypertensive medication therapy.

**Table 2.** Estimated Percentage Point Difference in Nonadherence to Antihypertensive Medications by Overall Population and Food Security Status

Study population	Nonadherence to antihypertensive medications^a^		PATE, percentage point difference (95% CI)^b^
	SNAP receipt	No SNAP receipt	
Overall	687 (56.4)	3162 (55.8)	1.7 (−21.2 to 24.6)
Food insecure	302 (56.0)	491 (60.0)	−13.6 (−25.0 to −2.3)
Food secure	385 (56.7)	2671 (55.2)	5.0 (−15.8 to 25.7)

Abbreviations: PATE, population average treatment effect; SNAP, Supplemental Nutrition Assistance Program.

^a^ Values are presented as No. (%) of participants, with unweighted No. values and weighted percentages given.

^b^ Estimated PATE values are the average marginal effects from a logit model and show the percentage point difference in nonadherence to antihypertensive medications between SNAP recipients (vs non-SNAP recipients) with hypertension. All regressions include a constant and all other covariates from Table 1 and are weighted by a product of the propensity score weight and the survey weight.

### Potential Dose-Response Associations Between Frequency of SNAP Receipt and Medication Nonadherence

**Figure 3.**
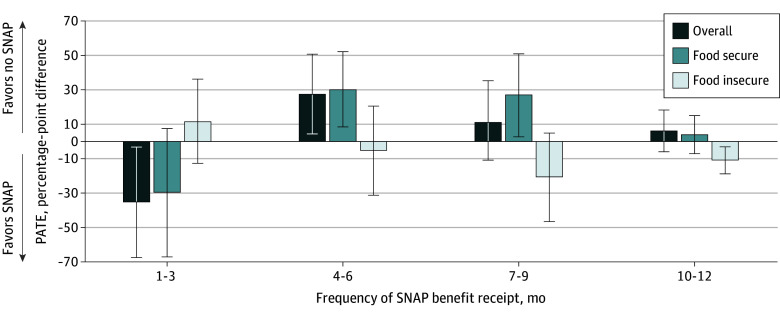
Estimated Population Average Treatment Effects (PATEs) of the Associations Between Levels of Frequency of Supplemental Nutrition Assistance Program (SNAP) Benefit Receipt and Nonadherence to Antihypertensive Medications Error bars indicate 95% CIs.

We observed 2 slightly contrasting U-shaped associations for the food-insecure subgroup and for the food-secure subgroup and the overall population. Although nonadherence was substantially lower among those who received SNAP 1 to 3 times per year, compared with nonrecipients, among the overall population, no associations were found among the food-insecure and food-secure subgroups. However, the direction of the association reversed when comparing receipt of SNAP 4 to 6 times per year vs no receipt; this frequency of SNAP receipt was associated with substantially higher nonadherence for the overall population and the food-secure subgroup but not for the food-insecure subgroup. A similar form of association was observed for the third-highest level of SNAP receipt. However, the highest frequency of SNAP recipient (10-12 times per year) vs no SNAP receipt was associated with lower rates of nonadherence among only the food-insecure subgroup (PATE, −11.0% [95% CI, −18.9% to −3.0%]) (Figure 3).

## Discussion

Among a US sample of patients with hypertension identified from a linked, nationally representative dataset, we observed that SNAP had a differential association with nonadherence to antihypertensive medications between food-secure and food-insecure subgroups. Specifically, individuals who received SNAP had a lower rate of nonadherence to antihypertensive medications among the food-insecure subgroup but not among the food-secure subgroup or the overall population. These findings suggest that SNAP may potentially mitigate against the risk of nonadherence to antihypertensive medications due to food insecurity. Our study contributes to the growing literature on how existing social intervention programs such as SNAP may be leveraged to prevent negative consequences of health-related social needs on health outcomes, including medication adherence.

To our knowledge, no published studies have specifically evaluated the role of food insecurity as a potential effect modifier of the associations between SNAP and adherence to antihypertensive medications among a nationally representative sample of insured and uninsured adults in the US. However, a few published studies have evaluated the overall associations between SNAP and medication adherence, including antihypertensive medications.^17,37,38^ In one analysis of NHIS data involving older adults (aged ≥60 years), researchers observed decreases in cost-related medication adherence (defined as delaying or skipping medications, or both, due to cost).^37^ Compared with nonrecipients, SNAP recipients were less likely to report cost-related medication nonadherence among the overall population (PATE, −4.8 [95% CI, −1.9 to −7.7]) and among a subgroup of NHIS participants who reported experiencing food insecurity (PATE, −7.4 [95% CI, −0.3 to −14.5]).^37^ Although that study was conducted among only older adults and was not focused on antihypertensive medications, the magnitude of the association of SNAP receipt with cost-related medication nonadherence was similar to what we observed in our study. In a similar analysis of the NHIS data involving only low-income older adults with diabetes (aged ≥65 years), SNAP participation was associated with a moderate decrease in cost-related medication nonadherence (PATE, −5.3 [95% CI, −10.0 to −0.5]).^17^ In a recent analysis of data from older adult Medicaid beneficiaries from Missouri, researchers reported that SNAP was positively associated (β = 0.32; *P* < .001) with MRA to antihypertensive medications in the overall population^38^; however, the role of food insecurity was not evaluated in that study. Although all 3 of the aforementioned studies^17,37,38^ reported that SNAP participation was associated with lower medication nonadherence, this was not the case in our analysis. The previous studies^17,37,38^ used a cross-sectional study design and limited the analysis to older adults, in contrast with our use of a retrospective longitudinal study design involving all eligible adults who reported having hypertension and were taking antihypertensive medications. Nonetheless, taken together, findings from these 3 studies suggest that SNAP participation may potentially increase medication adherence, especially cost-related medication nonadherence.

Our findings have several clinical and policy implications. Given the recent rollbacks in SNAP assistance, more individuals and families are likely to experience food insecurity and may be less likely to refill medications to treat chronic disease.^21,39,40^ The results of our sensitivity analysis suggested that SNAP may be associated with higher adherence for even the overall antihypertensive user population that was included. Therefore, these findings suggest that policy makers should expand SNAP access among patients with hypertension to prevent nonadherence to antihypertensive medications. Clinicians may also help increase adherence by identifying and connecting food-insecure patients with hypertension to food assistance programs provided by community-based social organizations. Our findings also suggest that SNAP participation may potentially increase medication adherence in general and not just cost-related nonadherence, considering that we controlled for health insurance status and out-of-pocket spending as suggested by previous studies.^37^ If our findings are confirmed by other studies, especially through interventional or quasi-experimental designs, it is possible that the benefits of SNAP may well go beyond addressing cost-related barriers.

### Strengths and Limitations

Our study has several strengths. First, unlike in prior studies, our study population included individuals with and without health insurance, using a nationally representative linked dataset. As such, our findings are more generalizable to US patients with hypertension. Second, this study presented data suggesting that the potential consequences of SNAP receipt on adherence to antihypertensive medications are greatest among the food-insecure population. Thus, we have identified the food-insecure hypertension population as a target for food assistance intervention to address adherence to antihypertensive medications, especially when food assistance resources are limited. Third, we leveraged the panel data structure of the MEPS to implement a longitudinal study design that allowed us to delineate and ensure that the period for assessment of SNAP exposure and food insecurity preceded the follow-up period for the assessment of adherence.

Our study also has limitations that must be taken into account when interpreting our findings. First, the observational study design limits any causal interpretation of the findings. Nonetheless, given that SNAP receipt was reported prior to the observation period for assessment of adherence, we were able to rule out potential reverse associations between SNAP and adherence. The causal-inference approach applied to investigate the associations in this study also further bolstered the associations observed against the potential effects of measured confounders. Second, MRA is based on records of filled prescriptions, which may be different from actual patient consumption of these medications. Third, our analysis assumed that all individuals who reported receiving SNAP benefits used the vouchers to purchase food. However, there are existing concerns about SNAP fraud, whereby some individuals trade SNAP vouchers for money and other goods and services. Fourth, because SNAP receipt was not randomized, it is possible that our findings may have been influenced by unmeasured confounding.

## Conclusions

The findings of this cohort study suggest that patients with hypertension who receive SNAP benefits may be less likely to become medication nonadherent, especially if they are experiencing food insecurity. We therefore recommend further examination of the role of SNAP as a potential intervention for preventing nonadherence to antihypertensive medications through prospectively designed interventional studies or natural experiment study designs.

## Notes

Supplement 1. **eAppendix 1.** Data Sources and Data Integration **eFigure.** Flowchart of Sample Selection for the Analysis **eAppendix 2.** Inverse Probability of Weighting (IPW) to Control Confounding Factors and Regression Specification **eTable.** List of Antihypertensive Agents by Therapeutic Class Codes
eReferences

Supplement 2.
Data Sharing Statement
