# Supplementary material for: Supplemental Nutrition Assistance Program and Adherence to Antihypertensive Medications
Source: JAMA Netw Open. 2024 Feb 23;7(2):e2356619. doi: 10.1001/jamanetworkopen.2023.56619 (PMC10891466; doi:10.1001/jamanetworkopen.2023.56619)
Supplement: Supplement 1. — eAppendix 1. Data Sources and Data Integration eFigure. Flowchart of Sample Selection for the Analysis eAppendix 2. Inverse Probability of Weighting (IPW) to Control Confounding Factors and Regression Specification eTable. List of Antihypertensive Agents by Therapeutic Class Codes eReferences [file jamanetwopen-e2356619-s001.pdf]

Supplementary Online Content

Islam MM, Oyarzun Gonzalez X, Bose-Brill S, Donneyong MM. Evaluation of the association between the Supplemental Nutrition Assistance Program and adherence to antihypertensive medications. *JAMA Netw Open*. 2024;7(2):e2356619. doi:10.1001/jamanetworkopen.2023.56619

eAppendix 1. Data Sources and Data Integration
eFigure. Flowchart of Sample Selection for the Analysis
eAppendix 2. Inverse Probability of Weighting (IPW) to Control Confounding Factors and Regression Specification
eTable. List of Antihypertensive Agents by Therapeutic Class Codes
eReferences

This supplementary material has been provided by the authors to give readers additional information about their work.

eAppendix 1. Data Sources and Data Integration

The primary aim of this study is to determine the correlation between SNAP participation and the probability of adhering to antihypertensive medication. To assess the effect of SNAP on medication adherence, we linked two datasets: the 2016-2017 Medical Expenditure Survey Panel and the National Health Interview Survey. The AHRQ Data Center provides access to all MEPS-NHIS Link files completed between 1996 and 2021. Each of these files includes a crosswalk, allowing users to merge MEPS Full-Year public use files (PUFs) with NHIS person-level public use data files. These NHIS files contain information collected from MEPS respondents in the year prior to their initial year of participation in MEPS. The reason for linking these datasets is that SNAP recipient status is available in the NHIS data, while food security status is present in the MEPS data. For an overview of the two datasets, refer to the following chart:

**Features of data**	**Medical Expenditure Survey Panel (MEPS)**	**National Health Interview Survey (NHIS)**
Years	1996-2019	1996 – 2019
Design	Longitudinal surveys	Cross-sectional
Participants	Families and individuals, medical providers (doctors, hospitals, pharmacies, etc.) and employers	Members of households
Innovation	Prescription medication use, food security (2016 -2017), healthcare access and satisfaction with care, health status and medical conditions, medical expenditure	Use of SNAP, health status, healthcare access, health conditions, health behaviors, immunization, etc.,
Link	https://meps.ahrq.gov/mepsweb/	https://www.cdc.gov/nchs/nhis/data-questionnaires-documentation.htm

© 2024 Islam MM et al. *JAMA Network Open.*

The Medical Expenditure Panel Survey (MEPS) Household Component (HC) survey utilizes the National Health Interview Survey (NHIS) as its sampling frame. Annually, a fresh MEPS-HC panel is formed, drawing from the previous year's NHIS sample. The MEPS-HC employs an overlapping panel design, where each household provides data for two consecutive calendar years through in-person interviews conducted over five rounds, with Round 3 covering both calendar years.

The MEPS full-year public use files cover the calendar year of the data year and contain data from rounds 3, 4, and 5 of the MEPS first panel (which uses the NHIS prior data year 1 as its sampling frame) combined with data from rounds 1, 2, and 3 of the MEPS second panel (which uses the NHIS prior data year 2 as its sampling frame). As presented in the below graphic, for full calendar year 2017 estimates, rounds 3, 4, and 5 of Panel 21 (which uses the 2015 NHIS as its sampling frame) are combined with rounds 1, 2, and 3 of Panel 22 (which uses the 2016 NHIS as its sampling frame).

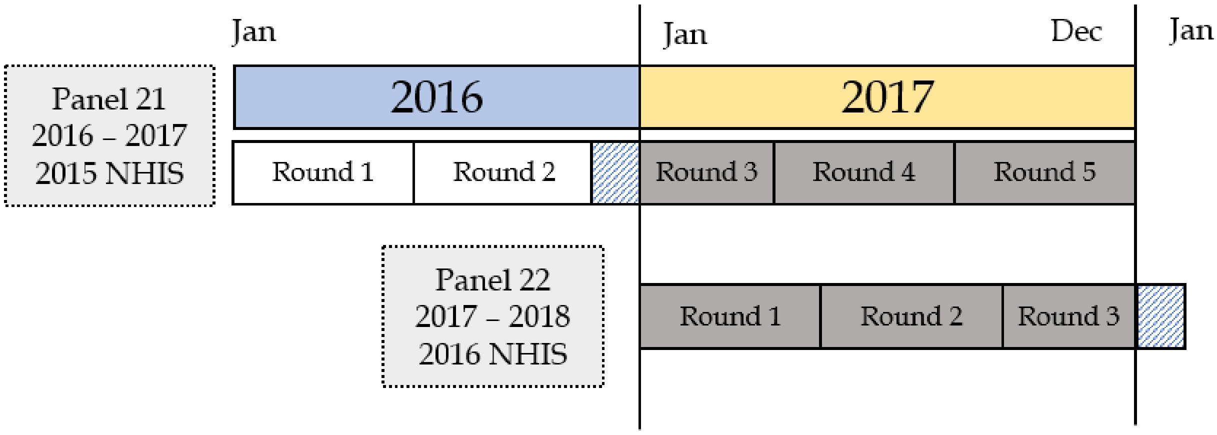

© 2024 Islam MM et al. *JAMA Network Open.*

eFigure. Flowchart of Sample Selection for the Analysis

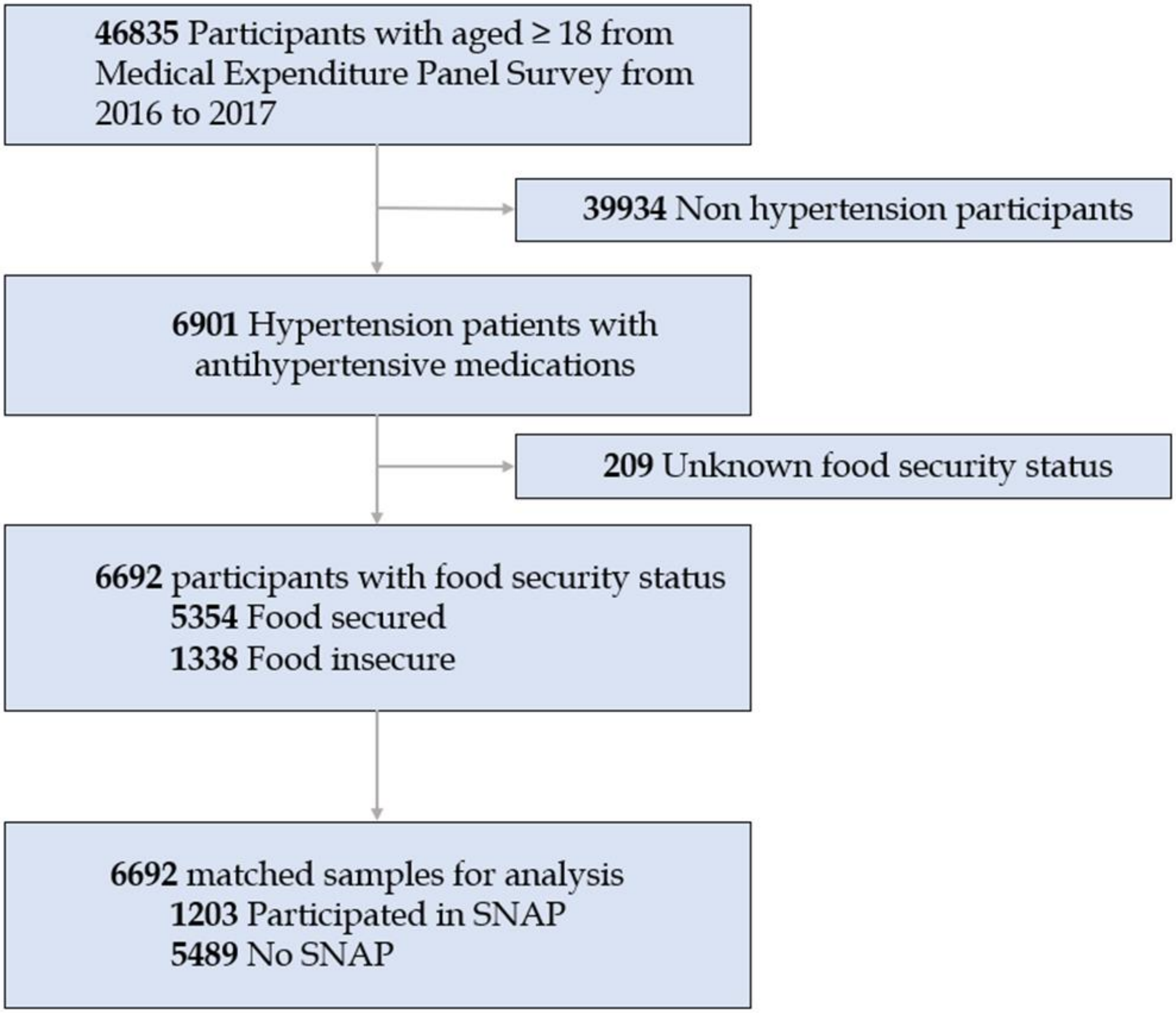

© 2024 Islam MM et al. *JAMA Network Open.*

eAppendix 2. Inverse Probability of Weighting (IPW) to Control Confounding Factors and Regression Specification

The objective of our study is to manage confounding factors that can influence the overall effect. Researchers have developed various statistical approaches to assess the relationship between the intervention and the desired outcome while controlling for confounders. Although these traditional methods are effective in obtaining unbiased estimates of causal effects, there are challenges that analysts need to be mindful of, as they might compromise the accuracy of their estimates.

In this study, we employ the inverse probability of weighting (IPW) approach to mitigate the impact of confounders. The IPW method consists of two main steps:

a) Generating propensity scores: We calculate the probability or propensity of an individual being exposed to the intervention based on their characteristics. The propensity score (PS) is used to calculate each individuals weight:
For treated/exposed individuals: weight = 1/PS
For untreated/unexposed individuals: weight = 1/(1-PS)
b) Applying weights: Each individual is assigned weights equal to the inverse of the probability of them receiving the actual exposure level. This process creates a pseudo dataset, where each observation is weighted by a function of the inverse propensity score. Consequently, the confounding variables no longer have a relationship with the treatment of interest in the pseudo dataset [1,2].

In this study, we employed a weighted probit regression model to assess the population average treatment effects (PATE) of SNAP on adherence to antihypertensive medications. To ensure the comparability between the two groups, and to estimate the PATE that can be generalized to the target population[3], we incorporated the survey weight in both stages of our analysis. In the first step, we included the survey weight as a predictor in the propensity score model. This step helps account for any relevant factors not already captured by other covariates, such as variables related to an individual's probability of responding to the survey. In the second stage, the regression was weighted by the product of the propensity score weight and survey weight. This approach further ensures that the estimation of the treatment effect is appropriately adjusted for both the participation probability and survey weight, leading to more reliable and robust results.

Influence of confounders in the outcome:

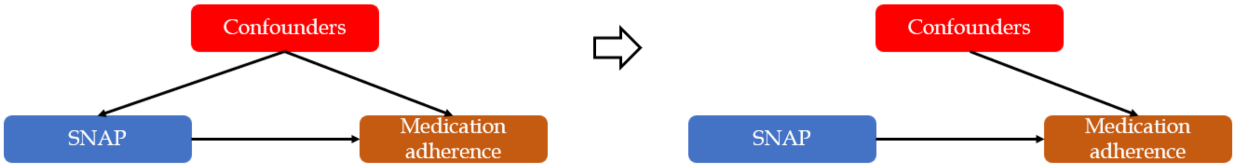

© 2024 Islam MM et al. *JAMA Network Open.*

eTable. List of Antihypertensive Agents by Therapeutic Class Codes

Therapeutic Class Code	Definition	Drug class
56	angiotensin II inhibitors	Angiotensin II receptor antagonists (ARBs)
42	angiotensin converting enzyme inhibitors	Angiotensin-converting enzyme inhibitors (ACEIs)
43	antiadrenergic agents, peripherally acting	Antiadrenergics
44	antiadrenergic agents, centrally acting	Antiadrenergics
47	beta-adrenergic blocking agents	Antiadrenergics
274	cardioselective beta blockers	Beta blockers (BBs)
275	non-cardioselective beta blockers	Beta blockers (BBs)
48	calcium channel blocking agents	Calcium channel blockers (CCBs)
55	antihypertensive combinations	Combined antihyp products
467	ace inhibitors with thiazides	Combined antihyp products
468	antiadrenergic agents (central) with thiazides	Combined antihyp products
469	antiadrenergic agents (peripheral) with thiazides	Combined antihyp products
470	miscellaneous antihypertensive combinations	Combined antihyp products
472	beta blockers with thiazides	Combined antihyp products
473	angiotensin II inhibitors with thiazides	Combined antihyp products
474	beta blockers with calcium channel blockers	Combined antihyp products
475	potassium sparing diuretics with thiazides	Combined antihyp products
476	ace inhibitors with calcium channel blocking agents	Combined antihyp products
479	angiotensin II inhibitors with calcium channel blockers	Combined antihyp products
482	angiotensin receptor blockers and neprilysin inhibitors	Combined antihyp products
49	diuretics	Diuretics
154	loop diuretics	Diuretics
155	potassium-sparing diuretics	Diuretics
156	thiazide diuretics	Diuretics
340	aldosterone receptor antagonists	Diuretics
342	renin inhibitors	Other renin-angiotensin system (RAS) antagonists
41	agents for hypertensive emergencies	Others
53	vasodilators	Vasodilators

© 2024 Islam MM et al. *JAMA Network Open.*

eReferences

1. Mansournia MA, Altman DG. Inverse probability weighting. *Bmj*. 2016;352
2. Seaman SR, White IR. Review of inverse probability weighting for dealing with missing data. *Statistical methods in medical research*. 2013;22(3):278-295.
3. DuGoff EH, Schuler M, Stuart EA. Generalizing observational study results: applying propensity score methods to complex surveys. *Health services research*. 2014;49(1):284-303.

© 2024 Islam MM et al. *JAMA Network Open.*
