# Supplementary material for: Supplemental Nutrition Assistance Program and Adherence to Antihypertensive Medications
Source: JAMA Netw Open. 2024 Feb 23;7(2):e2356619. doi: 10.1001/jamanetworkopen.2023.56619 (PMC10891466; doi:10.1001/jamanetworkopen.2023.56619)
Supplement: Supplement 2. — Data Sharing Statement [file jamanetwopen-e2356619-s002.pdf]

Data Sharing Statement

Islam. Supplemental Nutrition Assistance Program and Adherence to Antihypertensive Medications. *JAMA Netw Open*. Published February 23, 2024. doi:10.1001/jamanetworkopen.2023.56619

Data
Data available: No
